# Refining the Feasibility of Machine‐Learning‐Based Diagnostic Model Utilizing Gut Microbiota Analysis for Colorectal Cancer Screening

**DOI:** 10.1002/cam4.70935

**Published:** 2025-07-03

**Authors:** Shintaro Okumura, Yusuke Konishi, Taku Kitano, Tomonori Matsumoto, Kazutaka Obama, Satoshi Nagayama, Eiji Hara

**Affiliations:** ^1^ Research Institute for Microbial Diseases (RIMD) Osaka University Suita Japan; ^2^ Graduate School of Medicine, Kyoto University Kyoto Japan; ^3^ The Cancer Institute, Japanese Foundation for Cancer Research (JFCR) Tokyo Japan; ^4^ Uji‐Tokushukai Medical Center Uji Japan

**Keywords:** colorectal cancer, gut microbiota analysis, machine‐learning algorithm, screening

## Abstract

**Background:**

Recently, we developed a colorectal cancer (CRC) diagnostic model based on a machine learning algorithm with gut microbiota analysis. In this study, we evaluated the reproducibility of the diagnostic accuracy of the gut microbiota model, compared the diagnostic accuracy of the gut microbiota model with that of the fecal immunochemical test (FIT), and investigated the practical application potential of the gut microbiota model.

**Methods:**

Fecal samples were collected from both CRC patients and healthy individuals (HI) who underwent FIT. Gut microbiota analysis was performed using the same pipeline as that used in our previous study. Study subjects were diagnosed using the machine‐learning‐based gut microbiota model (ml‐GMM) with the same cut‐off value as in our previous study and by FIT.

**Results:**

The true positive rates of ml‐GMM and FIT were 53.1% and 86.4%, respectively, among 81 CRC patients, whereas the false positive rates among 245 HI cases were 7.3% and 2.4%, respectively. Evaluation of the proportion of either ml‐GMM or FIT being positive revealed a rate of 91.4% among CRC patients (Stage 0/I 78.3%; Stage II, 95.5%; Stage III, 96.6%; stage IV, 100.0%), whereas it was 9.4% among HI. Furthermore, we demonstrated a possible synergistic effect of ml‐GMM with FIT for detection of more CRC patients.

**Conclusions:**

The reproducibility of the diagnostic accuracy of ml‐GMM was confirmed. It was suggested that ml‐GMM in combination with FIT could detect more CRC patients than FIT alone.

## 1 Introduction

Colorectal cancer (CRC) is the third most common cancer and the second leading cause of cancer death in the world [1]. The annual global incidence and mortality rates are predicted to increase by 63% to 3.2 million cases and 73.4% to over 1.6 million cases, respectively, over the next two decades [2]. Therefore, its countermeasure is a global issue. The fecal immunochemical test (FIT) is a prevalent non‐invasive CRC screening method. The annual repetition of fecal tests has been proven to be effective in reducing CRC mortality in several randomized and controlled studies [3, 4, 5, 6]. However, CRC is detected in only 2%–3% [7, 8] of FIT‐positive individuals. More importantly, it has been reported that approximately 10%–20% of CRC patients cannot be detected by FIT screening [9, 10]. Although total colonoscopy is a more precise CRC screening method, it is invasive, expensive, and labor‐intensive. Therefore, the development of a reliable, non‐invasive CRC screening method is urgently needed.

Recently, we developed a CRC diagnostic model based on a machine learning algorithm with gut microbiota analysis [11]. Gut microbiota of a training dataset composed of 120 CRC patients and 120 healthy individuals (HI) were analyzed using H_2_O AutoML (https://www.H2O.ai/), an open‐source machine learning platform, to develop a diagnostic model that distinguishes CRC patients from HI. The diagnostic accuracy of the machine‐learning based gut microbiota model (ml‐GMM) was assessed with a test dataset composed of 426 CRC patients and 482 HI. When the cut‐off value of ml‐GMM was set so that the false positive rate was 7% in the training dataset, 45.8% of Stage I CRC patients and more than 60% of Stage II or higher CRC patients in the test dataset were diagnosed as positive, while 88.4% of HI were diagnosed as negative. In addition, 26 of the 34 HI with false‐positive FIT results were diagnosed as negative. Therefore, it was suggested that ml‐GMM might be a useful CRC screening method that supplements FIT. We were prompted to proceed with further study.

In this study, fecal samples were collected from a new population including CRC patients and HI who underwent concurrent FIT. The study subjects were diagnosed by ml‐GMM with the same cut‐off value as that of the previous study [11], as well as by FIT. Here, we report the results and discuss the possibility of practical usage of ml‐GMM in combination with FIT.

## 2 Methods

### 2.1 Fecal Sample Collection

Feces were collected from study participants who visited the Cancer Institute Hospital of the Japanese Foundation for Cancer Research (JFCR) (Tokyo) between April 2021 and January 2022 using a fecal sampling tool (TechnoSuruga Laboratory). FIT was simultaneously performed using Nescauto Hemo Plus (Alfresa Pharma Corporation) [10]. All study participants underwent a colonoscopy at the time of stool collection. Colorectal cancer patients (CRC patients) were defined as those with primary malignant epithelial colorectal tumors according to the Third English Edition of the Japanese Classification of Colorectal, Appendiceal, and Anal Carcinoma [12]. Healthy individuals (HI) were defined as those without colorectal cancer or advanced colorectal adenoma. HI were classified into two groups: clean HI who had no colorectal adenomas and HI with colorectal polyps smaller than 10 mm in size. We excluded patients with a history of inflammatory bowel disease, prior gastrointestinal reconstructive surgery, severe liver dysfunction (chronic hepatitis or liver cirrhosis), anticancer and/or antibiotic treatment within 1 month, stool collection within 3 days after colonoscopy, and failure to undergo FIT. Patients who received chemotherapy, radiation therapy, or colonic stent placement before fecal sample collection, who had fecal samples collected after endoscopic resection of tumors, or patients whose tumors were not primary colorectal tumors (e.g., squamous cell carcinoma of the anal canal cancer, metastasis, or direct invasion of other cancers to the large intestine) were also excluded. HI with a history of colorectal cancer, or with malignant tumors other than colorectal cancer, or abnormal endoscopic findings such as enteritis and hamartomas at the time of stool collection were excluded. Written informed consent was obtained from all participants for the use of anonymized samples and publication of the patients' clinical information under the protocol approved by the ethics committee of the JFCR hospital. The tumor profiles of CRC patients were classified based on the Third English Edition of the Japanese Classification of Colorectal, Appendiceal, and Anal Carcinoma [12].

### 2.2 16S rRNA Gene Sequencing Analysis and Microbiome Analysis

Bacterial DNA extraction from fecal samples was performed using a GENE STAR PI‐480 automated DNA isolation system (Kurabo Industries Ltd., Osaka, Japan). Polymerase chain reaction (PCR) amplification of the V1–V2 region of the bacterial 16S rRNA gene was performed using KAPA HiFi Hot Start Ready Mix (Roche) with universal 16S rRNA primers, followed by secondary amplification by adding Illumina flow cell adapters and indices. The PCR primers used are listed in Table S1. Meta‐16S rRNA gene sequencing was carried out per 192 samples on the Illumina MiSeq platform (Illumina Inc.) using MiSeq Reagent Kit v2 (Illumina Inc.) (paired‐end, 250 cycles × 2). These processes were performed by Biken Biomics Inc. Sequencing reads were processed using the QIIME2 (version 2020.8) pipeline [13]. Fastq files were de‐noised with the DADA2 plugin [14] and amplicon sequence variants (ASVs) were counted. Open‐reference clustering based on operational taxonomic units (OTUs) detected in the training data of the previous study [11] was performed. The OTUs of the training data in the previous study [11] were generated by de novo clustering using the VSEARCH plugin [15], with a similarity of more than 99%. OTU counts were converted to relative abundance per sample. A phylogenetic tree was generated from the ASVs, and beta diversity analyses (principal coordinate analyses of weighted UniFrac distance) were performed with a sampling depth of 10,000 reads.

### 2.3 Model Diagnosis

We previously calculated the sample size to maximize the diagnostic accuracy of ml‐GMM, leading to the estimation that 120 healthy individuals and 120 CRC patients were necessary in the training dataset [11]. To construct ml‐GMM, the H_2_O AutoML function with 10‐fold cross‐validations in the H_2_O package of R (version 3.32.0.1) (https://www.H2O.ai/) was performed. After repeating this process 10 times, the StackedEnsemble_BestOfFamily model with the highest diagnostic accuracy was selected as ml‐GMM [11]. In the diagnosis of each sample, OTUs with 99% similarity were used as variables for ml‐GMM. If the score of ml‐GMM is greater than 0.7194783, the sample is judged as positive.

### 2.4 Diagnostic Accuracy

The diagnostic accuracy of ml‐GMM and FIT was assessed with the true positive rate and the false positive rate. The true positive rate was defined as the rate of the test‐positive population in CRC patients. The false positive rate was defined as the rate of the test‐positive population in HI.

### 2.5 Statistical Analysis

Statistical analysis was performed using R (version 4.2.2). The differences in the characteristics of the samples and the α‐diversity in the study population were analyzed using a two‐tailed Wilcoxon rank sum test or a Fisher's exact test. The differences in scores of the CRC diagnostic model with gut microbiota analysis between groups were analyzed using a two‐tailed Wilcoxon rank‐sum test. Differences in diagnostic sensitivity were analyzed using a McNemar test. Differences were considered statistically significant when the *p* value was lower than 0.05.

## 3 Results

### 3.1 Recruitment of Study Participants

**FIGURE 1:**
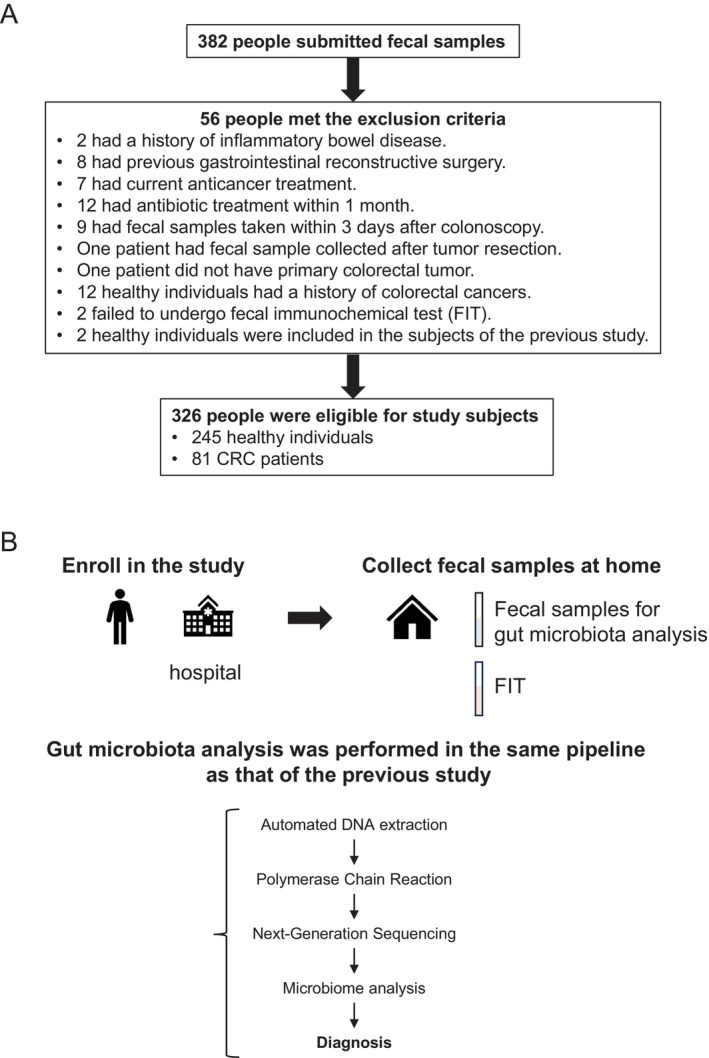
Study design. (A) Workflow chart for enrolling study subjects. A total of 382 individuals were enrolled in the study, and their fecal samples were collected. 54 people met the exclusion criteria set in the previous study or failed to undergo a fecal immunochemical test (FIT), and two healthy individuals were enrolled in the previous study. A total of 326 participants were included in this study. (B) Schema of the study design. Two fecal samples were collected at home from the participants enrolled in the study at the hospital. One was for gut microbiota analysis and the other was for FIT. The samples were sent to the laboratory. Gut microbiota analysis was performed using the same pipeline as that used in the previous study.

To explore the feasibility of ml‐GMM for practical use, fecal samples were collected from 382 participants. In this study, participants were required to undergo FIT simultaneously when collecting fecal samples for gut microbiota analysis. Among 382 people, 54 met the exclusion criteria set in the previous study [11] or failed to undergo FIT; two HI were already enrolled in the previous study [11]. Then, 81 CRC patients and 245 HI were subjected to analysis (Figure 1A, Table 1). In the current study, gut microbiota analysis was performed using the same pipeline as that used in the previous study [11] (Figure 1B). To examine the stability of the gut microbiota analysis in this pipeline, the *α* and *β* diversity of the gut microbiota in the current study and those in the previous study [11] were compared (Figure S1A,B).

**TABLE 1:** Clinical characteristics of study subjects.

	HI (*n* = 245)	CRC (*n* = 81)	*p*
Mean age (years old)	60	62	0.5
Sex: male/female	138/107	39/42	0.25
Mean BMI	23.5	22.7	0.81
Alcohol drinking: yes/no	179/56*	46/35	0.0016
Smoking: yes/no	26/209*	13/67*	0.24
Medications			
Proton pump inhibitor	20	10	0.27
Probiotics	4	6	0.018
Aspirin/NSAIDs	11	5	0.56
Anticoagulants	5	0	0.34
Immune suppressive agents	2	1	1
Metformin	7	3	0.71
Diabetes drug other than metformin	15	7	0.45
Personal history			
Diabetes	19	10	0.26
Hypertension	65	18	0.47
Hyperlipidemia	55	6	0.0018
Hyperuricemia	30	4	0.091
Autoimmune diseases	12	4	1
Endocrine organ diseases	22	4	0.34
Cholecystectomy	8	4	0.5
Appendectomy	34	8	0.45
Chronic liver diseases	7	2	1
Abdominal surgery without GI reconstruction	37	8	0.27
Malignant tumor	48	4	0.0014
Colorectal polyp	105	2	< 0.00001

*Note:* Statistical significance was determined using a two‐tailed Wilcoxon rank sum test (age and BMI) or a Fisher's exact test (sex, alcohol drinking, smoking, medications, and personal histories).

* Data of some study subjects was missing.

### 3.2 The Reproducibility of the Diagnostic Accuracy of Ml‐GMM

**FIGURE 2:**
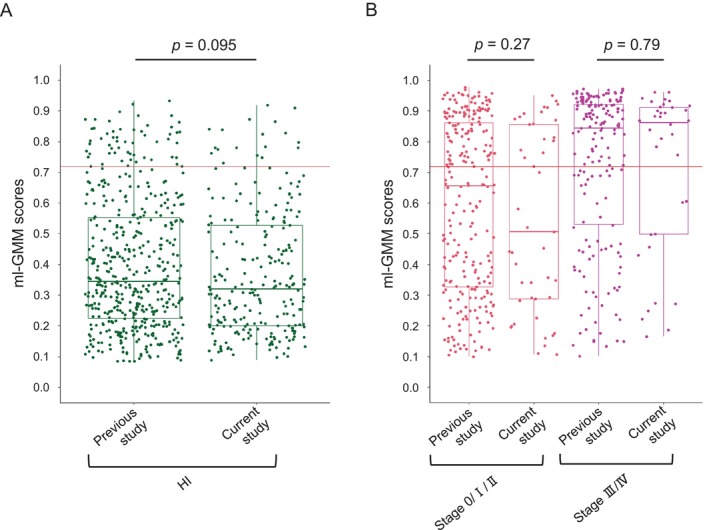
The reproducibility of the diagnostic accuracy of ml‐GMM. (A) The distributions of ml‐GMM scores in healthy individuals (HI) in the previous study (*n* = 482) and the current study (*n* = 245) were compared. (B) The distributions of ml‐GMM scores in stage 0/I/II CRC patients in the previous study (*n* = 252) and the current study (*n* = 45), and in stage III/IV CRC patients in the previous study (*n* = 174) and the current study (*n* = 36) were compared. Each dot represents the sample score. The boxes in the graph represent 25–75th percentiles, bold lines indicate the median, and whiskers extend to the maximum and minimum values within 1.5× the interquartile range. The red horizontal line indicates the cut‐off value of ml‐GMM at 0.7194783. Statistical significance was determined using the two‐tailed Wilcoxon rank‐sum test. *p* < 0.05 were considered significant (A, B).

We then assessed the reproducibility of the diagnostic accuracy of ml‐GMM. The distributions of model scores of HI, CRC patients in the early stage (Stage 0/I/II), and CRC patients in the late stage (Stage III/IV) were almost the same between the two studies, except that the model scores of HI with polyps in the current study were significantly lower than those in the previous study [11] (Figures 2A,B and S2A–C). Next, we compared the diagnostic accuracy of ml‐GMM with that of FIT. The cut‐off value of ml‐GMM was the same as that in the previous study [11]. The FIT cut‐off value was 100 ng/mL. The true positive rates of CRC patients in ml‐GMM and FIT were 53.1% and 86.4%, respectively, while the false positive rates of HI were 7.3% and 2.4%, respectively (*p* < 0.0001) (Table 2).

**TABLE 2:** Positive rate of ml‐GMM, FIT, and either of ml‐GMM or FIT in clean HI, HI with polyps, and patients with CRC in different stages.

	ml‐GMM positive	FIT positive	Either positive	ml‐GMM vs. FIT	FIT vs. Either
HI (*n* = 245)	7.3% (4.4–11.4)	2.4% (0.9–5.1)	9.4% (60.4–13.8)		
Clean HI (*n* = 158)	7.6% (4.0–12.9)	1.9% (0.4–5.4)	9.5% (5.4–15.2)		
HI with polyps (*n* = 87)	6.9% (2.6–14.4)	3.4% (0.7–9.7)	9.2% (4.1–17.3)		
CRC patients (*n* = 81)	53.1% (41.7–64.3)	86.4% (77.0–93.0)	91.4% (83.0–96.5)	*p* < 0.0001	*p* = 0.074
Stage 0/I (*n* = 23)	30.4% (13.2–52.9)	69.6% (47.1–86.8)	78.3% (56.3–92.5)		
Stage II (*n* = 22)	54.5% (32.2–75.6)	90.9% (70.8–98.9)	95.5% (77.2–99.9)		
Stage III (*n* = 29)	62.1% (42.3–79.3)	93.1% (77.2–99.2)	96.6% (82.2–99.9)		
Stage IV (*n* = 7)	85.7% (42.1–99.6)	85.7% (42.1–99.6)	100.0% (59.0–100.0)		

*Note:* The ranges in parentheses are 95% confidence intervals calculated using a binomial distribution. Statistical significance was determined using a McNemar test.

### 3.3 High CRC Detection Rate With the Combination of Ml‐GMM and FIT

**FIGURE 3:**
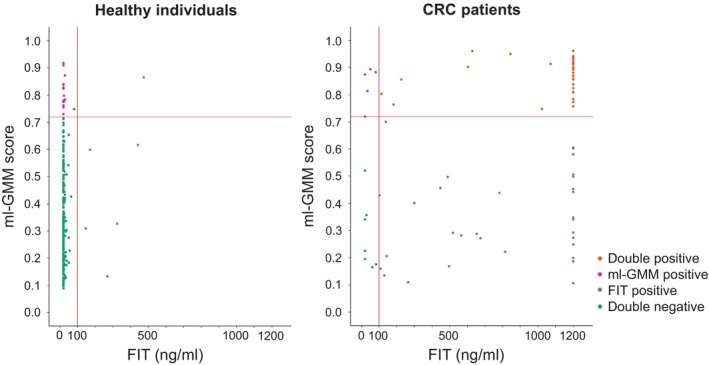
Comparison and combination of the diagnostic accuracy of ml‐GMM and FIT Scatterplots of ml‐GMM score and FIT value for each sample of healthy individuals (*n* = 245) and CRC patients (*n* = 81). Orange dots represent samples judged as positive by both ml‐GMM and FIT (double positive). The number was one in HI and 38 in CRC patients. The magenta dots represent samples judged to be positive only by ml‐GMM. The number of samples were 17 in HI and five in CRC patients. Purple dots represent samples judged to be positive only by FIT. The numbers of samples were five in HI and 31 in CRC patients. Green dots represent samples judged as negative by both ml‐GMM and FIT (double‐negative). The numbers of samples were 222 in HI and seven in CRC patients. The red horizontal line indicates the cut‐off value of ml‐GMM at 0.7194783. The red vertical line indicates the cut‐off value of FIT at 100 ng/mL.

Although the diagnostic accuracy of ml‐GMM was inferior to that of FIT, it was almost the same as that in the previous study [11]. Five CRC patients were not judged as positive by FIT but by ml‐GMM. (Figure 3). Therefore, we explored the possibility that ml‐GMM supplements FIT in CRC screening. To this end, we assessed the diagnostic accuracy of the combination of ml‐GMM and FIT by examining the percentage of those positive for either of them (either positive) in CRC patients and HI. The proportion of CRC patients that were either positive was 91.4% (Stage 0/I 78.3%; Stage II, 95.5%; Stage III, 96.6%; and Stage IV, 100.0%), while the false‐positive rate in HI was 9.4% (Table 2). Notably, the proportion of either positive in Stage 0/I was 8.7% that was superior to that of FIT‐positive (Table 2) and that in proximal colon cancer was 7.7% that was superior to that of FIT‐positive (Table 3); hence, it might be possible that the combination of ml‐GMM and FIT detected more CRC patients as either positive than FIT alone.

**TABLE 3:** Positive rate of ml‐GMM, FIT, and either of the microbiota model or FIT in patients with proximal colon cancer, distal colon cancer, or rectal cancer in different stages.

	ml‐GMM positive	FIT positive	Either positive
Proximal colon (*n* = 26)	34.6% (17.2–55.7)	84.6% (65.1–95.6)	92.3% (74.9–99.1)
Stage 0/I/II (*n* = 17)	29.4% (10.3–56.0)	76.5% (50.1–93.2)	88.2% (63.6–98.5)
Stage III/IV (*n* = 9)	44.4% (13.7–78.8)	100.0% (66.3–100.0)	100.0% (66.3–100.0)
Distal colon (*n* = 22)	59.1% (36.4–79.3)	81.8% (59.7–94.8)	86.4% (65.1–97.1)
Stage 0/I/II (*n* = 13)	53.8% (25.1–80.8)	69.2% (38.6–90.9)	76.9% (46.2–95.0)
Stage III/IV (*n* = 9)	66.7% (29.9–92.5)	100.0% (66.4–100.0)	100.0% (66.4–100.0)
Rectum (*n* = 33)	63.6% (45.1–79.6)	87.9% (71.8–96.6)	93.9% (79.8–99.3)
Stage 0/I/II (*n* = 15)	46.7% (21.3–73.4)	93.3% (68.1–99.8)	93.3% (68.1–99.8)
Stage III/IV (*n* = 18)	77.8% (52.4–93.6)	83.3% (58.6–96.4)	94.4% (72.7–99.9)

*Note:* Ranges in parentheses are 95% confidence intervals calculated with binomial distribution.

### 3.4 Exploration of Factors That Could Affect Diagnostic Accuracy of ml‐GMM

**FIGURE 4:**
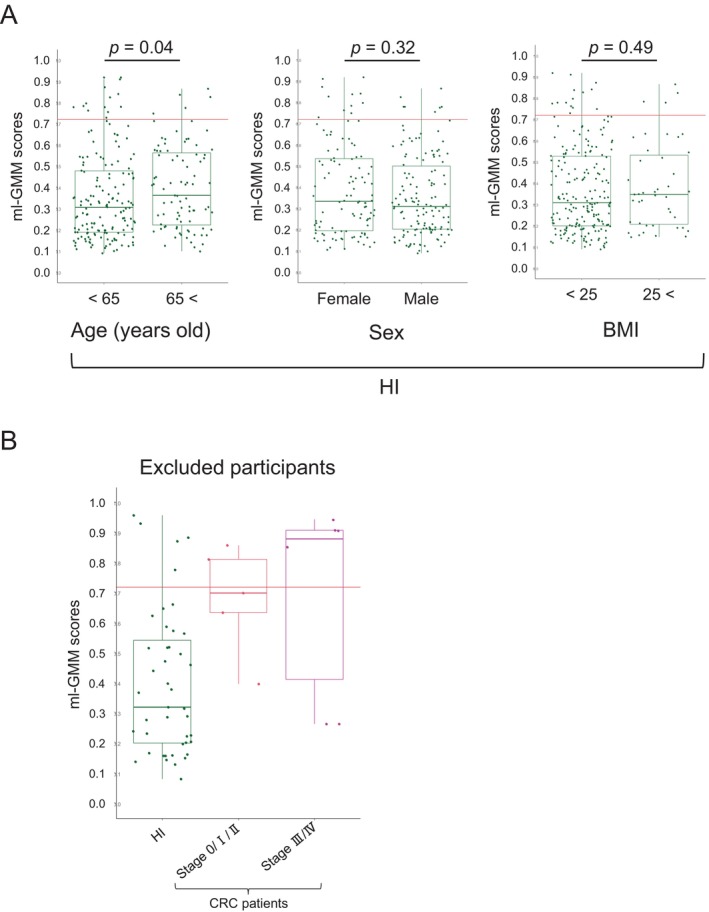
Exploration of factors that could affect diagnostic accuracy of ml‐GMM. (A) The distributions of scores of ml‐GMM in HI were compared by age (younger than 65 years of age: *n* = 158, 65 years of age or older: *n* = 87), sex (female: *n* = 107, male: *n* = 138), and body mass index (BMI) (< 25: *n* = 202, ≥ 25: *n* = 43). Statistical significance was determined with a two‐tailed Wilcoxon rank‐sum test. *p* < 0.05 were considered significant. (B) Distribution of scores of ml‐GMM in excluded participants (HI: *n* = 43, Stage 0/I/II CRC patients: *n* = 5, Stage III/IV CRC patients: *n* = 6).

Finally, we explored the factors that could affect the diagnostic accuracy of ml‐GMM. The distributions of the model scores in HI were comparable by sex (*p* = 0.32) and body mass index (BMI) (*p* = 0.49) (Figure 4A, Table S2). Model scores in HI younger than 65 years were significantly lower than those in HI aged 65 years or older (*p* = 0.04) (Figure 4A); the mean age was not significantly different between HI with false positives of ml‐GMM and HI with true negatives of ml‐GMM (*p* = 0.54). On the contrary, taking immunosuppressants (*p* = 0.0051), having autoimmune diseases (*p* = 0.0007), and having endocrine organ diseases (*p* = 0.014) were related to false positives in HI, although the number of HI with these factors was small (Table S2). Furthermore, we examined the diagnoses of the excluded 54 people (43 HI, five Stage 0/I/II CRC patients and six Stage III/IV CRC patients) who met the exclusion criteria or failed to undergo FIT. The distributions of the model scores of the excluded people were comparable with those of the study subjects in both HI and CRC patients (Figure 4B).

## 4 Discussion

In this study, ml‐GMM judged more than half of the CRC patients as positive with the same cut‐off value as that of the previous study [11], while the false‐positive rate was 7.3%. Although the diagnostic accuracy of ml‐GMM was not superior to that of FIT, it reproducibly distinguished CRC patients from HI. Remarkably, the combination of ml‐GMM and FIT diagnosed more than 90% of CRC patients as either positive, whereas the false‐positive rate of HI was below 10%. In particular, the combination of ml‐GMM and FIT improved the detection rates of Stage 0/I CRC patients and proximal colon cancer patients by more than 7%, compared with FIT alone. Therefore, the combination of ml‐GMM and FIT would be a non‐invasive CRC screening method with higher sensitivity than FIT alone.

Although the clinical efficacy of FIT, including the risk reduction of CRC mortality, has been established [3, 4, 5, 6], some people are likely to be judged as positive by FIT, even though no neoplastic lesions are detected with colonoscopy [16, 17]. Moreover, approximately half of Stage 0/I CRC patients were not detected by FIT [9]. Therefore, many types of biological markers have been targeted to devise more precise CRC screening methods. Recently, the diagnostic accuracy of a next‐generation multitarget fecal DNA test [18] and a cell‐free DNA blood test [19] for CRC screening has been reported in large‐population prospective studies. Both could detect over 80% of CRC patients, whereas their false positive rates were about 10%. These results suggest that the two novel CRC screening methods are suitable for practical use. Although the current study was a retrospective case–control study, the combination of ml‐GMM and FIT achieved almost the same diagnostic accuracy as that of the two screening methods. Therefore, ml‐GMM, in combination with FIT, is feasible for practical use.

It has long been expected that gut microbiota analysis will be a useful CRC screening method [20, 21, 22]. However, several technical problems interfere with its clinical application. In the previous study [11], we reported a stable diagnostic accuracy of ml‐GMM validated in an unknown test dataset with a fixed cut‐off value [11]. Because the data of gut microbiota analysis can be affected by experimental processes [23], the samples in the current study were processed in the same pipeline as that of the previous study [11]. In addition, considering the diversity of the gut microbiota composition between individuals, all bacterial taxa identified in the training dataset of the previous study were used as variables for ml‐GMM [11]. These well‐planned study designs would explain why our microbiota model could consistently work properly. Indeed, ml‐GMM could judge HI as negative, regardless of age, sex, and BMI. However, the proportions of taking immunosuppressants, having autoimmune diseases, and having endocrine organ diseases were possibly related to the false positives of HI. The composition of the gut microbiota can be affected by host immune surveillance or the endocrine system [24, 25, 26, 27, 28, 29]. Therefore, the diagnosis of the microbiota model might be inaccurate for people with disorders of immune function or the endocrine system. On the other hand, the distributions of model scores of the population meeting the exclusion criteria were comparable with those of study subjects in both HI and CRC patients. Thus, ml‐GMM might be also applicable for those with a history of inflammatory bowel disease, prior gastrointestinal reconstructive surgery, and severe liver dysfunction, although further study is needed to conclude it.

This study has some limitations. Firstly, this study was conducted in a single institution, whereas the previous study was conducted in three institutions [11]. Secondly, fecal samples of CRC patients were retrospectively collected after CRC diagnosis. Thirdly, we excluded some cases according to the exclusion criteria, which could impact the generalizability of the test. To consolidate the reliability and assess the generalizability of ml‐GMM, a prospective study with a larger sample size is required in parallel with the cost‐effectiveness analysis. In addition, given the increasing incidences of young onset of CRC and the fact that this population is not included in the screening paradigm in most countries, it would be desirable if the ability to detect CRC using our ml‐GMM analysis could extend to the young generation. However, there were only 14 CRC patients (6 of Stage 0/I and 8 of Stage II/III/IV) and 43 HI in this study. Hence, it would be difficult to discuss the applicability of ml‐GMM for a population younger than 50 in this study.

Nevertheless, it was confirmed that ml‐GMM could reproducibly diagnose CRC patients and HI with high accuracy with a fixed cut‐off value. Furthermore, it was suggested that the combination of ml‐GMM and FIT may be an improved CRC screening method that detects more CRC patients than FIT alone. This is the first report to suggest the practical use of a CRC diagnostic model with gut microbiota analysis.

## Author Contributions

E.H. and S.N. designed the experiments and oversaw the projects. S.O., Y.K., and T.K. performed meta 16S rRNA gene‐sequencing analysis and model diagnosis. S.O., T.M., K.O., S.N., and E.H. analyzed the data. S.O. visualized the data and wrote the manuscript. All the authors discussed the results and commented on the manuscript.

## Ethics Statement

Approval of the research protocol by an Institutional Review Board: The protocol for this research project has been approved by the Ethics Committee of the Cancer Institute Hospital of JFCR (Approval No. 2013‐1090) and it conforms to the provisions of the Declaration of Helsinki.

## Consent

Written informed consent was obtained from all participants for the use of anonymized samples and publication of the patients' clinical information.

## Conflicts of Interest

The authors declare no conflicts of interest.

## Supporting information


**Figure S1.** The diversity analysis of gut microbiota.
**Figure S2.** The reproducibility of the diagnostic accuracy of ml‐GMM in each subgroup.

**Table S1.** PCR primers used in this study.
**Table S2.** Clinical characteristics of HI with false positives and true.

## Data Availability

The data that support the findings of this study are available from the corresponding author upon reasonable request.

## References

[1] F. Bray , M. Laversanne , H. Sung , et al., “Global Cancer Statistics 2022: GLOBOCAN Estimates of Incidence and Mortality Worldwide for 36 Cancers in 185 Countries,” CA: A Cancer Journal for Clinicians 74 (2024): 21834, 10.3322/caac.21834. 38572751

[2] E. Morgan , M. Arnold , A. Gini , et al., “Global Burden of Colorectal Cancer in 2020 and 2040: Incidence and Mortality Estimates From GLOBOCAN,” Gut 72, no. 2 (2023): 338–344, 10.1136/gutjnl-2022-327736. 36604116

[3] A. Shaukat , S. J. Mongin , M. S. Geisser , et al., “Long‐Term Mortality After Screening for Colorectal Cancer,” New England Journal of Medicine 369 (2013): 1106–1114, 10.1056/NEJMoa1300720. 24047060

[4] J. H. Scholefield , S. M. Moss , C. M. Mangham , D. K. Whynes , and J. D. Hardcastle , “Nottingham Trial of Faecal Occult Blood Testing for Colorectal Cancer: A 20‐Year Follow‐Up,” Gut 61 (2012): 1036–1040, 10.1136/gutjnl-2011-300774. 22052062

[5] O. Kronborg , O. D. Jorgensen , C. Fenger , et al., “Randomized Study of Biennial Screening With a Faecal Occult Blood Test: Results After Nine Screening Rounds,” Scandinavian Journal of Gastroenterology 39 (2004): 846–851, 10.1080/00365520410003182. 15513382

[6] E. Lindholm , H. Brevinge , and E. Haglind , “Survival Benefit in a Randomized Clinical Trial of Faecal Occult Blood Screening for Colorectal Cancer,” British Journal of Surgery 95 (2008): 1029–1036, 10.1002/bjs.6136. 18563785

[7] H. Chen , J. Shi , M. Lu , et al., “Comparison of Colonoscopy, Fecal Immunochemical Test, and Risk‐Adapted Approach in a Colorectal Cancer Screening Trial (TARGET‐C),” Clinical Gastroenterology and Hepatology 21 (2023): 808–818, 10.1016/j.cgh.2022.08.003. 35964896

[8] M. Sekiguchi , M. Westerberg , C. Löwbeer , et al., “Endoscopist Adenoma Detection Rate Associated With Neoplasia Detection During Subsequent‐Round Colonoscopy in Fecal Immunochemical Test‐Based Colorectal Cancer Screening: Cross‐Sectional Analysis of the SCREESCO Randomized Controlled Trial,” Gastrointestinal Endoscopy 4 (2025): 16, 10.1016/j.gie.2025.01.037. 39914632

[9] T. Morikawa , J. Kato , Y. Yamaji , et al., “A Comparison of the Immunochemical Fecal Occult Blood Test and Total Colonoscopy in the Asymptomatic Population,” Gastroenterology 129, no. 2 (2005): 422–428. 16083699 10.1016/j.gastro.2005.05.056

[10] Y. Oono , Y. Iriguchi , Y. Doi , et al., “A Retrospective Study of Immunochemical Fecal Occult Blood Testing for Colorectal Cancer Detection,” Clinica Chimica Acta 411 (2010): 802–805. 10.1016/j.cca.2010.02.057 20184867

[11] Y. Konishi , S. Okumura , T. Matsumoto , et al., “Development and Evaluation of a Colorectal Cancer Screening Method Using Machine Learning‐Based Gut Microbiota Analysis,” Cancer Medicine 11 (2022): 3194–3206, 10.1002/cam4.4671. 35318827 PMC9385600

[12] Japanese Society for Cancer of the Colon and Rectum , “Japanese Classification of Colorectal, Appendiceal, and Anal Carcinoma: The 3d English Edition,” Journal of the Anus, Rectum and Colon 3 (2019): 175–195, 10.23922/jarc.2019-018. 31768468 PMC6845287

[13] E. Bolyen , J. R. Rideout , M. R. Dillon , et al., “Reproducible, Interactive, Scalable and Extensible Microbiome Data Science Using QIIME 2,” National Biotechnology 37, no. 8 (2019): 852–857, 10.1038/s41587-019-0209-9. PMC7015180 31341288

[14] B. J. Callahan , P. J. McMurdie , M. J. Rosen , A. W. Han , A. J. Johnson , and S. P. Holmes , “DADA2: High‐Resolution Sample Inference From Illumina Amplicon Data,” Nature Methods 13, no. 7 (2016): 581–583, 10.1038/nmeth.3869. 27214047 PMC4927377

[15] T. Rognes , T. Flouri , B. Nichols , C. Quince , and F. Mahé , “VSEARCH: A Versatile Open Source Tool for Metagenomics,” PeerJ 4 (2016): e2584, 10.7717/peerj.2584. 27781170 PMC5075697

[16] M. J. Domper Arnal , S. García Mateo , S. Hermoso‐Durán , et al., “False‐Positive Fecal Immunochemical Test Results in Colorectal Cancer Screening and Gastrointestinal Drug Use,” International Journal of Colorectal Disease 36, no. 9 (2021): 1861–1869, 10.1007/s00384-021-03947-1. 33982138

[17] E. L. Amitay , K. Cuk , T. Niedermaier , K. Weigl , and H. Brenner , “Factors Associated With False‐Positive Fecal Immunochemical Tests in a Large German Colorectal Cancer Screening Study,” International Journal of Cancer 144, no. 10 (2019): 2419–2427, 10.1002/ijc.31972. 30411799

[18] T. F. Imperiale , K. Porter , J. Zella , et al., “Next‐Generation Multitarget Stool DNA Test for Colorectal Cancer Screening,” New England Journal of Medicine 390, no. 11 (2024): 984–993, 10.1056/NEJMoa2310336. 38477986

[19] D. C. Chung , D. M. Gray, 2nd , H. Singh , et al., “A Cell‐Free DNA Blood‐Based Test for Colorectal Cancer Screening,” New England Journal of Medicine 390, no. 11 (2024): 973–983, 10.1056/NEJMoa2304714. 38477985

[20] J. P. Zackular , M. A. Rogers , M. T. Ruffin , et al., “The Human Gut Microbiome as a Screening Tool for Colorectal Cancer,” Cancer Prevention Research (Philadelphia, Pa.) 7 (2014): 1112–1121, 10.1158/1940-6207. 25104642 PMC4221363

[21] N. T. Baxter , M. T. Ruffin , M. A. Rogers , and P. D. Schloss , “Microbiota‐Based Model Improves the Sensitivity of Fecal Immunochemical Test for Detecting Colonic Lesions,” Genome Medicine 8, no. 37 (2016): 3, 10.1186/s13073-016-0290-3. 27056827 PMC4823848

[22] G. Zeller , J. Tap , A. Y. Voigt , et al., “Potential of Fecal Microbiota for Early‐Stage Detection of Colorectal Cancer,” Molecular Systems Biology 10 (2014): 766, 10.15252/msb.20145645. 25432777 PMC4299606

[23] R. Sinha , G. Abu‐Ali , E. Vogtmann , et al., “Assessment of Variation in Microbial Community Amplicon Sequencing by the Microbiome Quality Control (MBQC) Project Consortium,” Nature Biotechnology 35 (2017): 1077–1086. 10.1038/nbt.3981 PMC5839636 28967885

[24] J. L. Kubinak , C. Petersen , W. Z. Stephens , et al., “MyD88 Signaling in T Cells Directs IgA‐Mediated Control of the Microbiota to Promote Health,” Cell Host & Microbe 17, no. 2 (2015): 153–163, 10.1016/j.chom.2014.12.009. 25620548 PMC4451207

[25] J. J. Bunker , T. M. Flynn , J. C. Koval , et al., “Innate and Adaptive Humoral Responses Coat Distinct Commensal Bacteria With Immunoglobulin A,” Immunity 43, no. 3 (2015): 541–553, 10.1016/j.immuni.2015.08.007. 26320660 PMC4575282

[26] S. Kawamoto , K. Uemura , N. Hori , et al., “Bacterial Induction of B Cell Senescence Promotes Age‐Related Changes in the Gut Microbiota,” Nature Cell Biology 25, no. 6 (2023): 865–876, 10.1038/s41556-023-01145-5. 37169880

[27] M. J. Mendoza‐León , A. K. Mangalam , A. Regaldiz , et al., “Gut Microbiota Short‐Chain Fatty Acids and Their Impact on the Host Thyroid Function and Diseases,” Frontiers in Endocrinology 14 (2023): 1192216, 10.3389/fendo.2023.1192216. 37455925 PMC10349397

[28] Y. Sun , S. Gao , C. Ye , and W. Zhao , “Gut Microbiota Dysbiosis in Polycystic Ovary Syndrome: Mechanisms of Progression and Clinical Applications,” Frontiers in Cellular and Infection Microbiology 13 (2023): 1142041, 10.3389/fcimb.2023.1142041. 36909735 PMC9998696

[29] C. Virili , I. Stramazzo , and M. Centanni , “Gut Microbiome and Thyroid Autoimmunity,” Best Practice & Research. Clinical Endocrinology & Metabolism 35, no. 3 (2021): 101506, 10.1016/j.beem.2021.101506. 33648848

