# Supplementary material for: Refining the Feasibility of Machine‐Learning‐Based Diagnostic Model Utilizing Gut Microbiota Analysis for Colorectal Cancer Screening
Source: Cancer Med. 2025 Jul 3;14(13):e70935. doi: 10.1002/cam4.70935 (PMC12226176; doi:10.1002/cam4.70935)
Supplement: Supplementary file 1 — Figure S1. The diversity analysis of gut microbiota. Figure S2. The reproducibility of the diagnostic accuracy of ml‐GMM in each subgroup. [file CAM4-14-e70935-s002.pptx]

## Slide 1
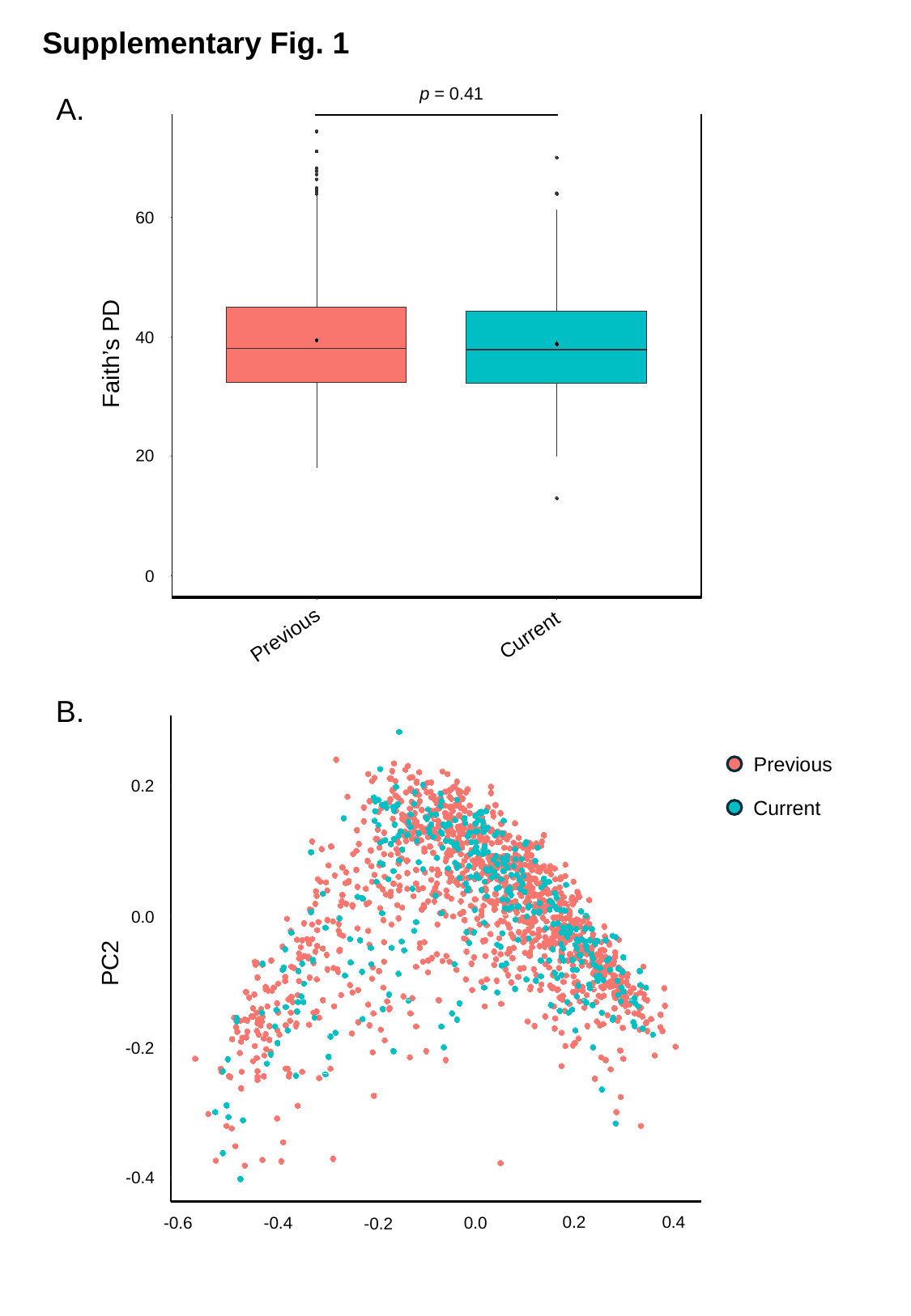

Supplementary Fig. 1
p = 0.41
A.
60
40
Faith’s PD
20
0
Previous
Current
B.
Previous
Current
0.2
0.0
PC2
-0.2
-0.4
0.2
0.4
-0.6
-0.4
0.0
-0.2

## Slide 2
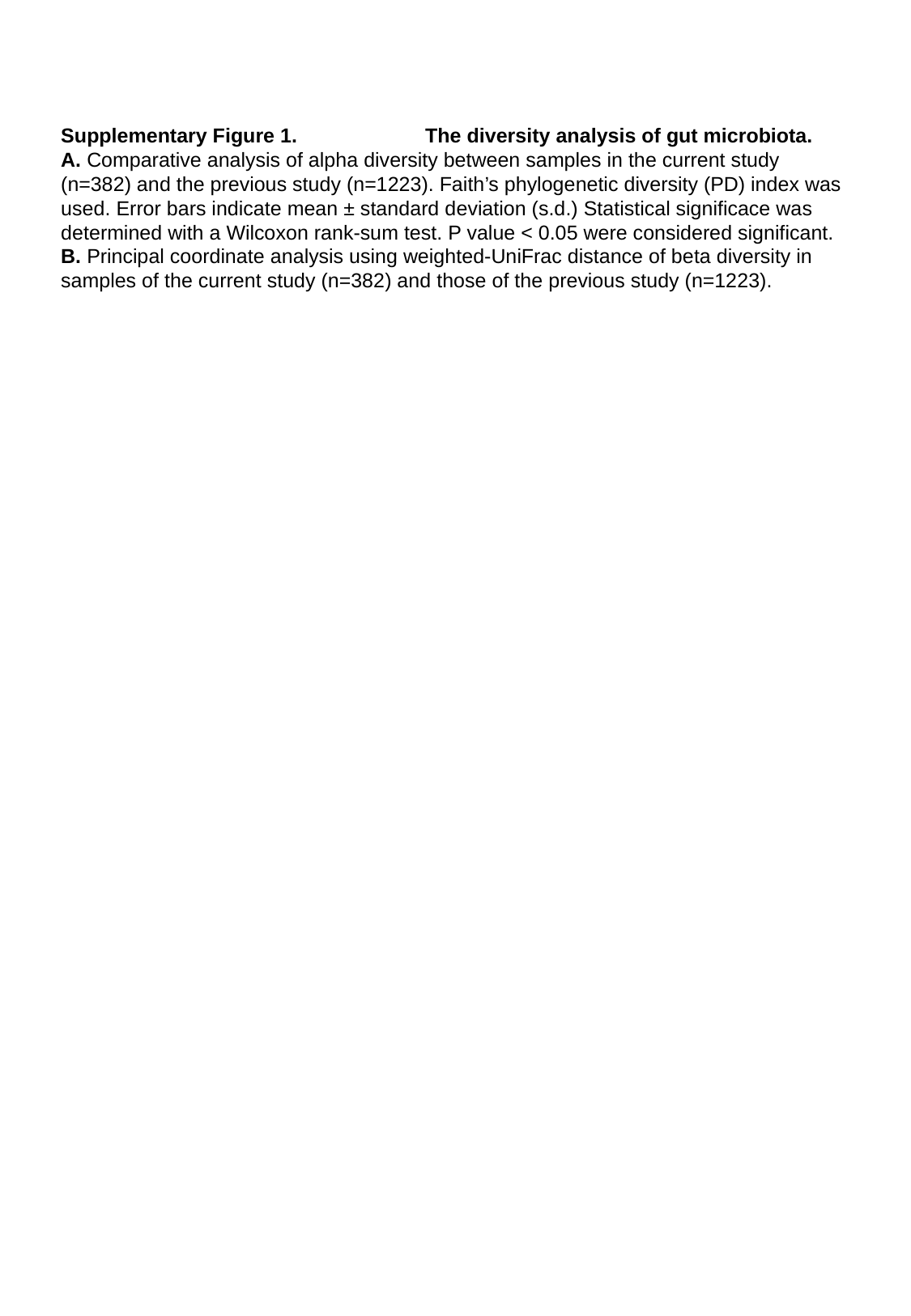

Supplementary Figure 1.		The diversity analysis of gut microbiota.
A. Comparative analysis of alpha diversity between samples in the current study (n=382) and the previous study (n=1223). Faith’s phylogenetic diversity (PD) index was used. Error bars indicate mean ± standard deviation (s.d.) Statistical significace was determined with a Wilcoxon rank-sum test. P value < 0.05 were considered significant.
B. Principal coordinate analysis using weighted-UniFrac distance of beta diversity in samples of the current study (n=382) and those of the previous study (n=1223).

## Slide 3
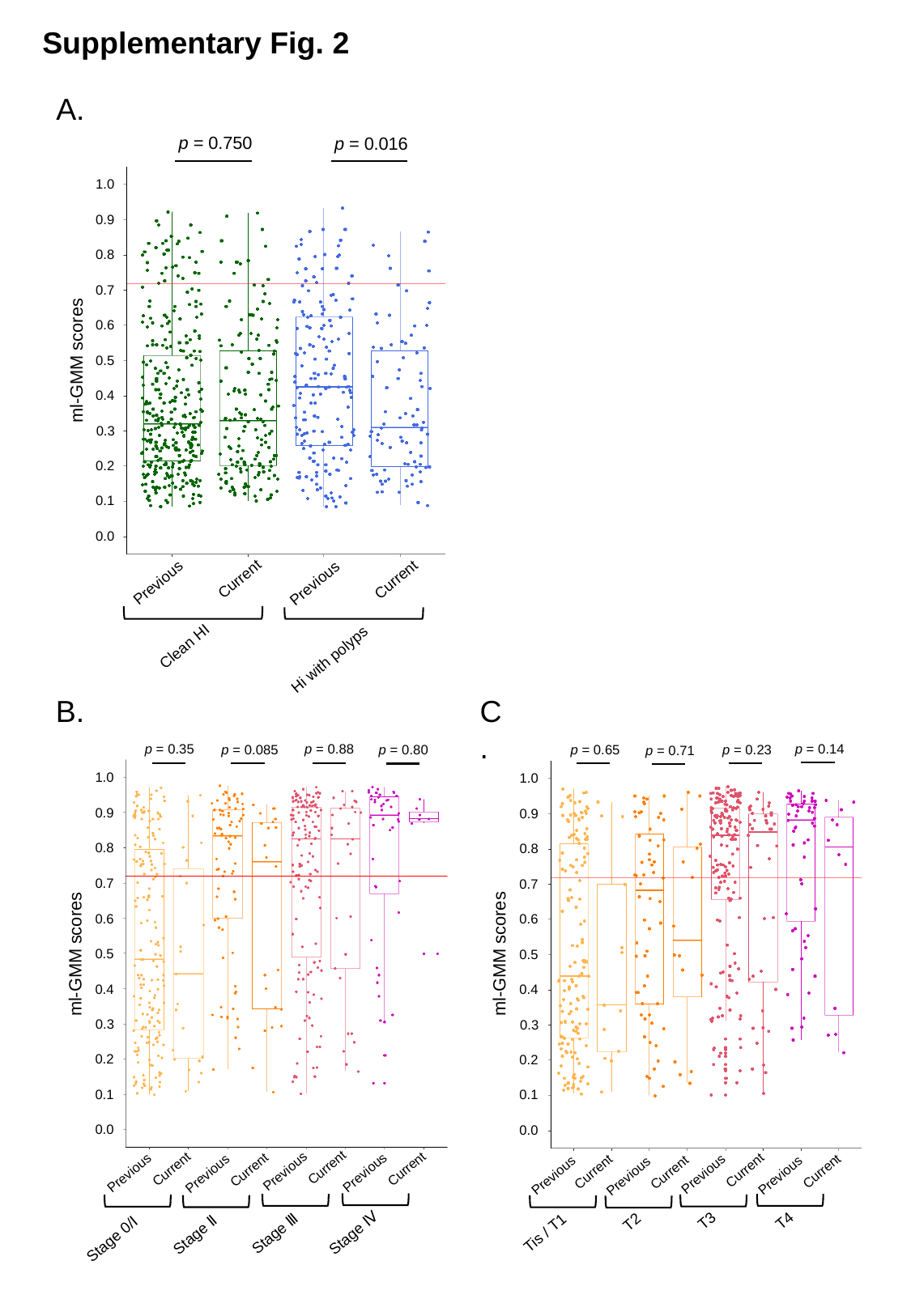

Supplementary Fig. 2
A.
p = 0.750
p = 0.016
1.0
0.9
0.8
0.7
0.6
0.5
0.4
0.3
0.2
0.1
0.0
Clean HI
Hi with polyps
Current
Current
Previous
Previous
ml-GMM scores
B.
C.
p = 0.88
p = 0.35
p = 0.80
p = 0.085
1.0
0.9
0.8
0.7
0.6
0.5
0.4
0.3
0.2
0.1
0.0
Stage Ⅳ
Stage Ⅲ
Stage Ⅱ
Stage 0/Ⅰ
Current
Current
Current
Current
Previous
Previous
Previous
Previous
p = 0.14
p = 0.65
p = 0.23
p = 0.71
1.0
0.9
0.8
0.7
0.6
0.5
0.4
0.3
0.2
0.1
0.0
T4
T3
T2
Tis / T1
Current
Current
Current
Current
Previous
Previous
Previous
Previous
ml-GMM scores
ml-GMM scores

## Slide 4
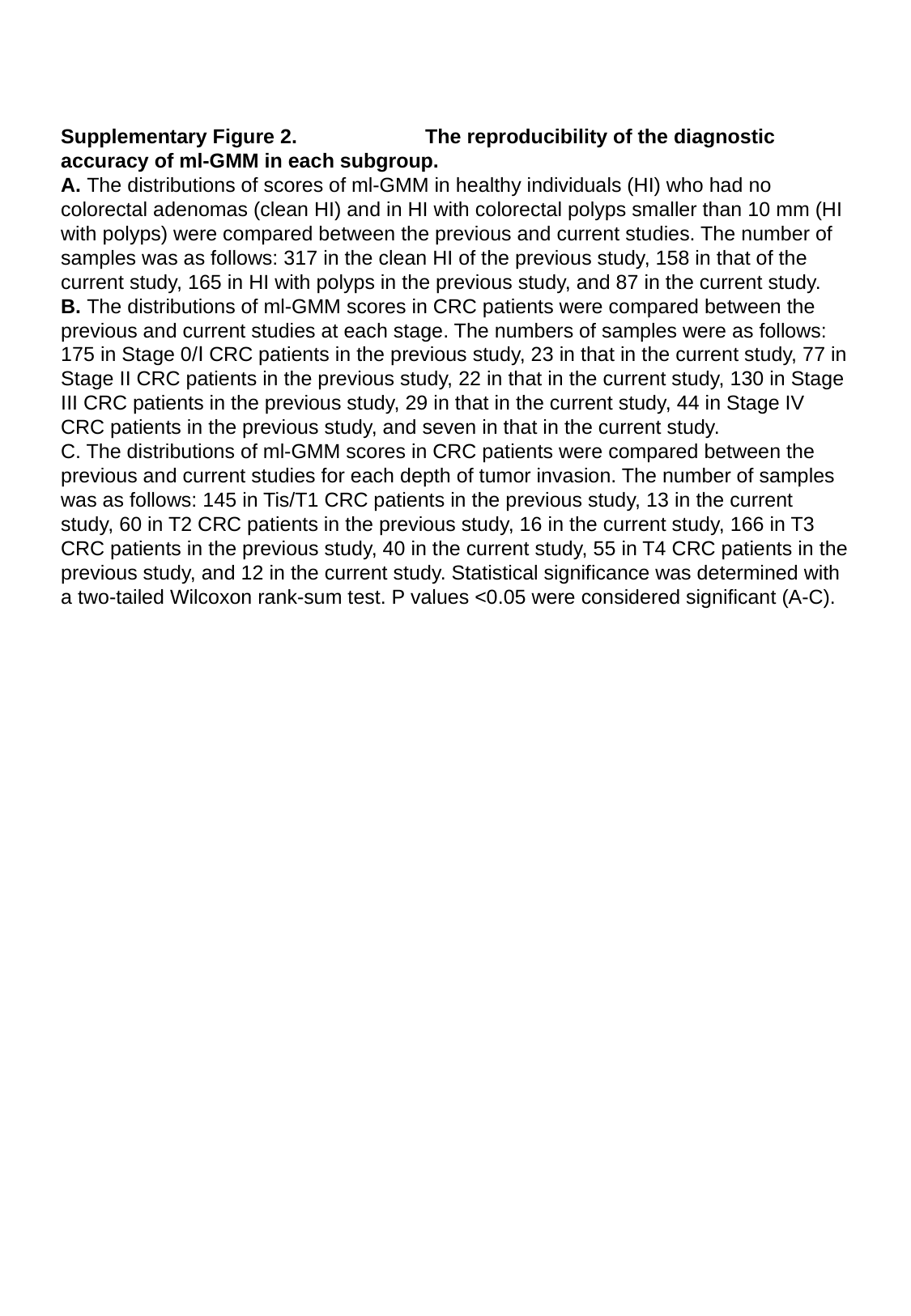

Supplementary Figure 2.		The reproducibility of the diagnostic accuracy of ml-GMM in each subgroup.
A. The distributions of scores of ml-GMM in healthy individuals (HI) who had no colorectal adenomas (clean HI) and in HI with colorectal polyps smaller than 10 mm (HI with polyps) were compared between the previous and current studies. The number of samples was as follows: 317 in the clean HI of the previous study, 158 in that of the current study, 165 in HI with polyps in the previous study, and 87 in the current study.
B. The distributions of ml-GMM scores in CRC patients were compared between the previous and current studies at each stage. The numbers of samples were as follows: 175 in Stage 0/Ⅰ CRC patients in the previous study, 23 in that in the current study, 77 in Stage II CRC patients in the previous study, 22 in that in the current study, 130 in Stage III CRC patients in the previous study, 29 in that in the current study, 44 in Stage IV CRC patients in the previous study, and seven in that in the current study.
C. The distributions of ml-GMM scores in CRC patients were compared between the previous and current studies for each depth of tumor invasion. The number of samples was as follows: 145 in Tis/T1 CRC patients in the previous study, 13 in the current study, 60 in T2 CRC patients in the previous study, 16 in the current study, 166 in T3 CRC patients in the previous study, 40 in the current study, 55 in T4 CRC patients in the previous study, and 12 in the current study. Statistical significance was determined with a two-tailed Wilcoxon rank-sum test. P values <0.05 were considered significant (A-C).
