# Supplementary material for: Refining the Feasibility of Machine‐Learning‐Based Diagnostic Model Utilizing Gut Microbiota Analysis for Colorectal Cancer Screening
Source: Cancer Med. 2025 Jul 3;14(13):e70935. doi: 10.1002/cam4.70935 (PMC12226176; doi:10.1002/cam4.70935)
Supplement: Supplementary file 2 — Table S1. PCR primers used in this study. Table S2. Clinical characteristics of HI with false positives and true. [file CAM4-14-e70935-s001.docx]

**Supplementary Table 1. PCR primers used in this study**

| **Primers for 16S rRNA gene-sequencing** | | |
| --- | --- | --- |
| **1^st^ PCR** | | |
| **Primer name** |  | **Sequence (overhangs are underlined)** |
| 16S-27Fmod | Forward | TCGTCGGCAGCGTCAGATGTGTATAAGAGACAGAGRGTTTGATYMTGGCTCAG |
| 16S-338R | Reverse | GTCTCGTGGGCTCGGAGATGTGTATAAGAGACAGTGCTGCCTCCCGTAGGAGT |
| **2^nd^ PCR** | | |
| Nextera DNA Indexes (Illumina Inc., San Diego, CA, USA) | | |

**Supplementary Table 2. Clinical characteristics of HI with false positives and true negatives**

|  | HI with the false positive  (n=18) | HI with the true negative  (n=227) | *P* value |
| --- | --- | --- | --- |
| **Average age (years old)** | 57 | 61 | 0.54 |
| **Sex  Male / Female** | 7/11/ | 131 / 96 | 0.14 |
| **Average BMI** | 22.2 | 22.5 | 0.9 |
| **Alcohol drinking Yes / No** | 12 / 6 / | 167 / 59 | 0.58 |
| **Smoking Yes / No** | 0 / 18 | 26 / 191 | 0.23 |
| **Medications** |  |  |  |
| Proton pump inhibitor | 2 | 18 | 0.65 |
| Probiotics | 0 | 4 | 1 |
| Aspirin / NSAIDs | 1 | 10 | 0.58 |
| Anticoagulants | 1 | 4 | 0.32 |
| Immunosuppressants | 2 | 0 | 0.0051 |
| Metformin | 0 | 14 | 0.61 |
| Diabetes drug other than metformin | 1 | 7 | 0.46 |
| **Personal history** |  |  |  |
| Diabetes | 1 | 18 | 1 |
| Hypertension | 5 | 60 | 1 |
| Hyperlipidemia | 2 | 53 | 0.38 |
| Hyperuricemia | 3 | 27 | 0.47 |
| Autoimmune diseases | 5 | 7 | 0.0007 |
| Endocrine organ diseases | 5 | 17 | 0.014 |
| Cholecystectomy | 0 | 8 | 1 |
| Appendectomy | 1 | 33 | 0.48 |
| Chronic liver diseases | 0 | 7 | 1 |
| Abdominal surgery without GI reconstruction | 3 | 34 | 0.74 |
| Malignant tumor | 4 | 44 | 0.76 |
| Colorectal polyp | 6 | 99 | 0.46 |

Statistical significance was determined with a two- tailed Wilcoxon rank sum test (age and BMI) or a Fisher exact test (sex, alcohol drinking, smoking, medications and personal histories).
